# Supplementary material for: Proactive and reactive accumulation-to-bound processes compete during perceptual decisions
Source: Nat Commun. 2021 Dec 8;12:7148. doi: 10.1038/s41467-021-27302-8 (PMC8655090; doi:10.1038/s41467-021-27302-8)
Supplement: Supplementary file 1 — Supplementary Information [file 41467_2021_27302_MOESM1_ESM.pdf]

# Proactive and reactive accumulation-to-bound processes compete during perceptual decisions

Lluís Hernández-Navarro, Ainhoa Hermoso-Mendizabal, Daniel Duque, Jaime de la Rocha and Alexandre Hyafil

## Supplementary Figures

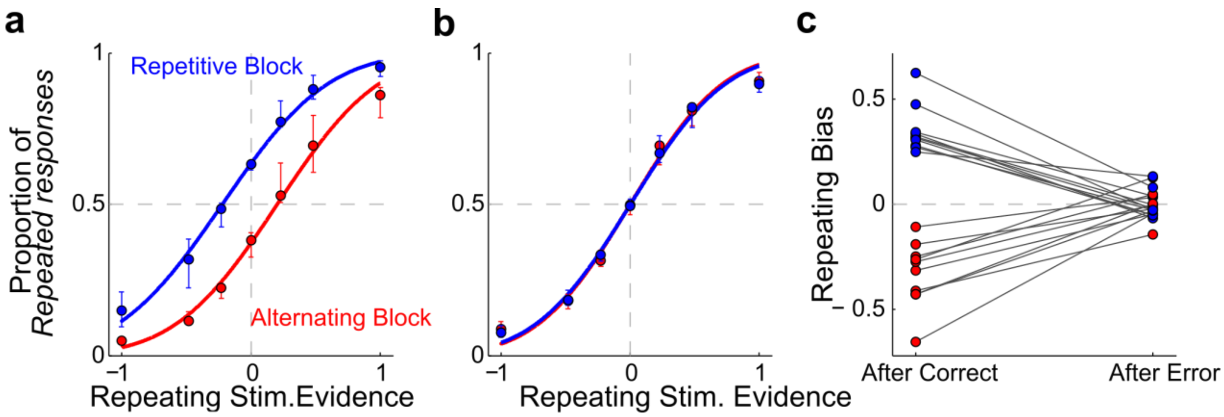


**Supplementary Figure 1. Repeating Bias reset after error trials. a-b,** Proportion of Repeated responses vs. Repeating stimulus evidence computed in the Repetitive (blue dots) or Alternating blocks (red dots; color code applies for all panels), and computed in trials following a correct (a) or an incorrect response (b). The repeating stimulus evidence shows the stimulus evidence in favor of repeating the previous choice (positive values: evidence to repeat; negative values: evidence to alternate); the absolute value of the repeating stimulus evidence corresponds to the stimulus strength *s*. Each point shows the median across Group 1 animals ($n$ = 10) and error bars show first and third quartiles. Curves show fits using a probit function. **c,** Repeating bias, defined as the horizontal shift in the psychometric curves shown in panels a-b, for each rat in each block after a correct or an error response. Each pair of connected dots represents one animal. All animals developed a block-dependent repeating bias after rewarded trials (expectation-biased trials). However, the bias vanished after an unrewarded trial in both types of block (unbiased trials) [1].

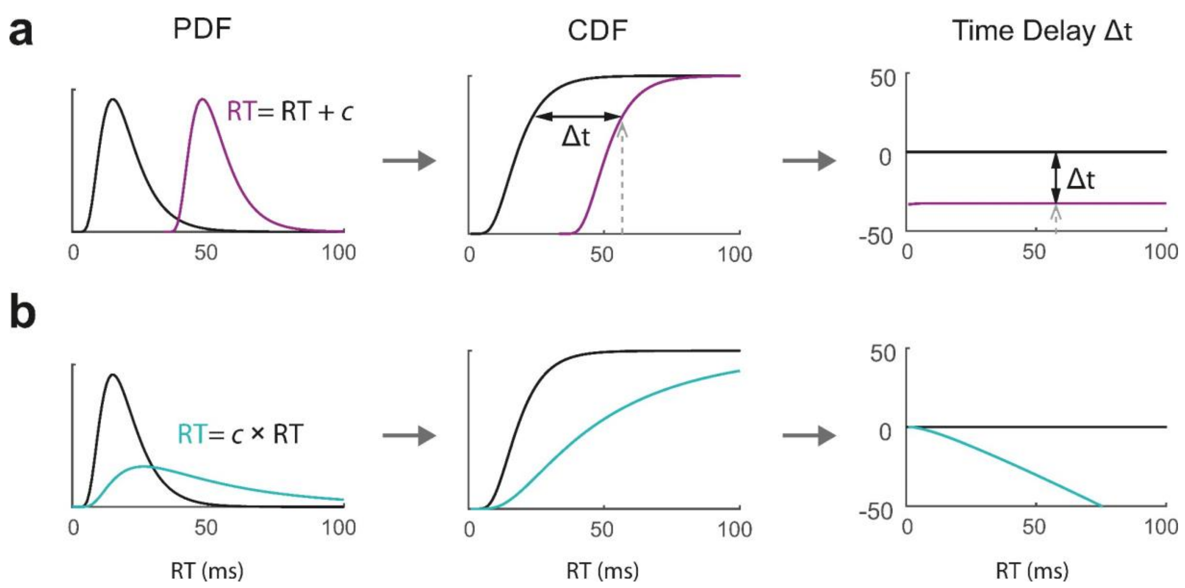


**Supplementary Figure 2. Time delay curves for exemplar reaction time distributions. a,** Additive modulation of reaction time (RT). Left: reference RT probability density function (pdf, black), and target RT pdf (purple) computed as the reference pdf slowed by a constant amount *c*. Center: corresponding cumulative distribution functions (cdfs); for each RT value, time delay $\Delta$t is defined as the temporal delay between target and reference cdfs at the corresponding value of the target cdf (vertical dashed gray arrow). Right: time delay curves set as the value of $\Delta$t as a function of RT, here equal to the constant $\Delta$t = -*c*. **b,** Multiplicative example. Left: same as in panel a, but for the target (cyan) computed as the multiplicative slowing of the reference pdf by a factor *c*. Center: same as in a. Right: same as in a, but with non-constant time delay $\Delta$t = -(*c*-1)·RT. Source data are provided as a Source Data file.

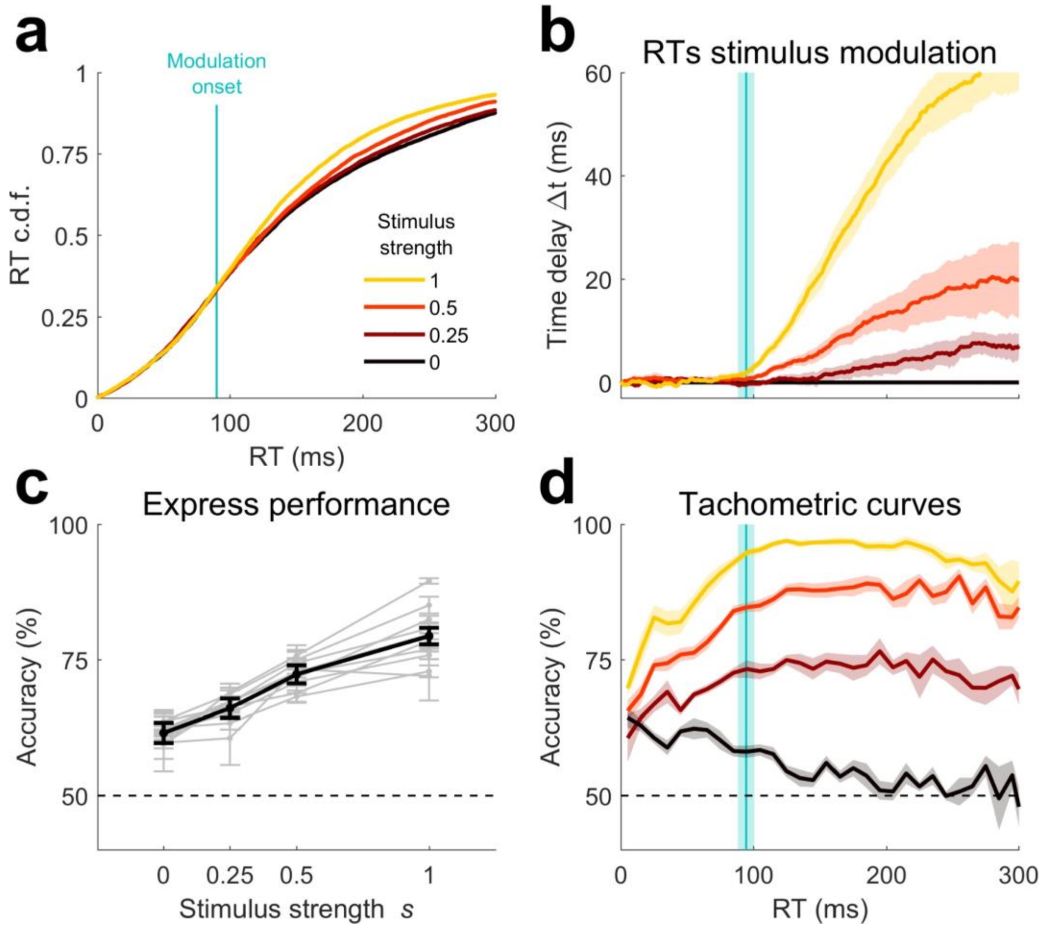


**Supplementary Figure 3. Decoupled reaction times and choices for express responses in biased trials.** Legend as in Fig. 2 of the main manuscript, but for expectation-biased trials (i.e. trials after rewarded responses; see Supplementary Fig. 1a). RT cumulative distribution functions (RT cdf, rat #14) (a), and time delay curves (b) for biased trials are qualitatively similar to those for unbiased trials (Fig. 2a-b). However, accuracy in express responses was higher for biased trials, and especially for most ambiguous stimuli ($s$=0), where accuracy was well above chance level (c). Moreover, accuracy was higher than chance even for RTs close to 0 ms, irrespective of stimulus strength (d). This reflected that rats capitalized the existence of serial correlations in the stimulus sequence and, in these biased trials, used their expectations to partially predict the upcoming stimulus [1]. All data points, data lines, error bars and error bands in panels b-d represent mean values ± s.e.m. across rats in Group 1 ($n$ = 10). Source data are provided as a Source Data file.

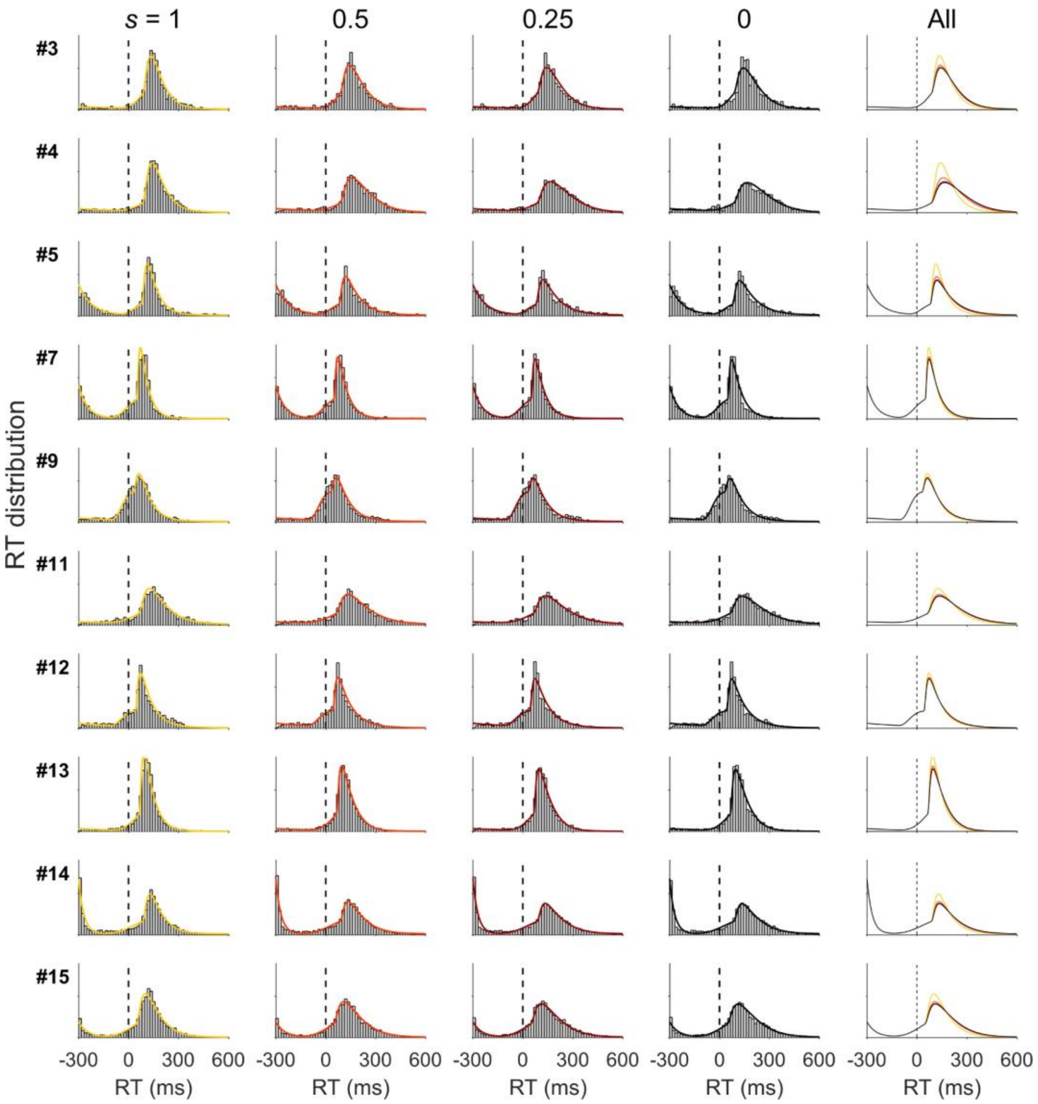


**Supplementary Figure 4. PSIAM fit to reaction times for unbiased trials.** RT distributions (gray bars) and model fits (color lines) for all rats in Group 1 (*n* = 10 rows, numeric label is rat index) and stimulus strength *s* (columns, colors) in unbiased, post-error trials. Last column: superposed model fit curves for all stimulus strengths. Vertical dashed lines denote stimulus onset. Each row represents one animal. Note the large fraction of early fixation breaks (from -300 to -150 ms), especially for rats #5, 7, 14 and 15. Source data are provided as a Source Data file.

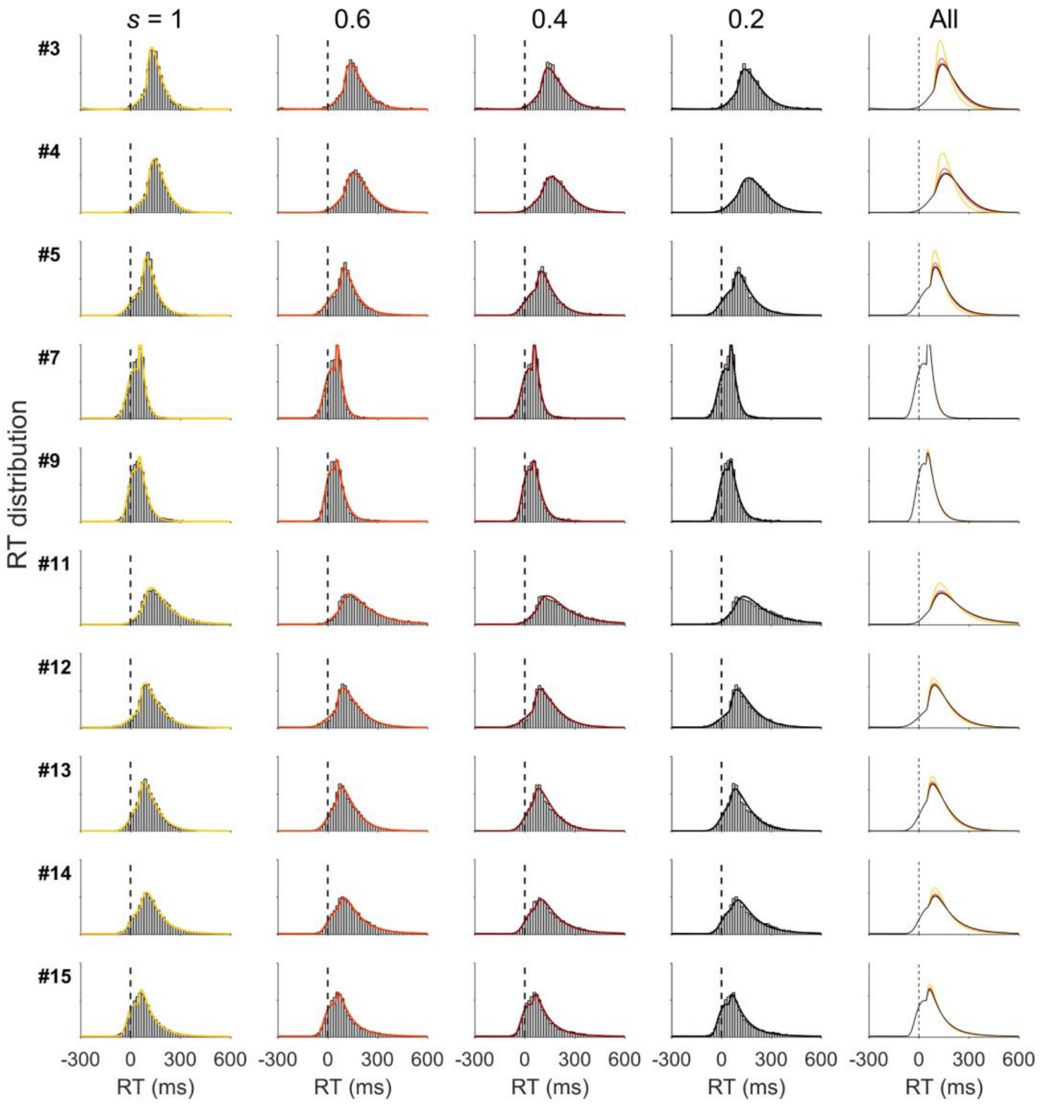


**Supplementary Figure 5. PSIAM fit to reaction times for biased trials.** RT distributions (gray bars) and model fits (color lines) for all rats in Group 1 (*n* = 10 rows, numeric label is rat index) and stimulus strengths *s* (columns, colors) in expectation-biased trials (i.e. after-correct trials). Last column: superposed model fit curves for all stimulus strengths for each rat. Vertical dashed lines denote stimulus onset. Each row represents one animal. Note the absence of early fixation breaks (from -300 to -150 ms) in biased, post-correct trials as opposed to unbiased, post-error trials (Supplementary Fig. 4). Source data are provided as a Source Data file.

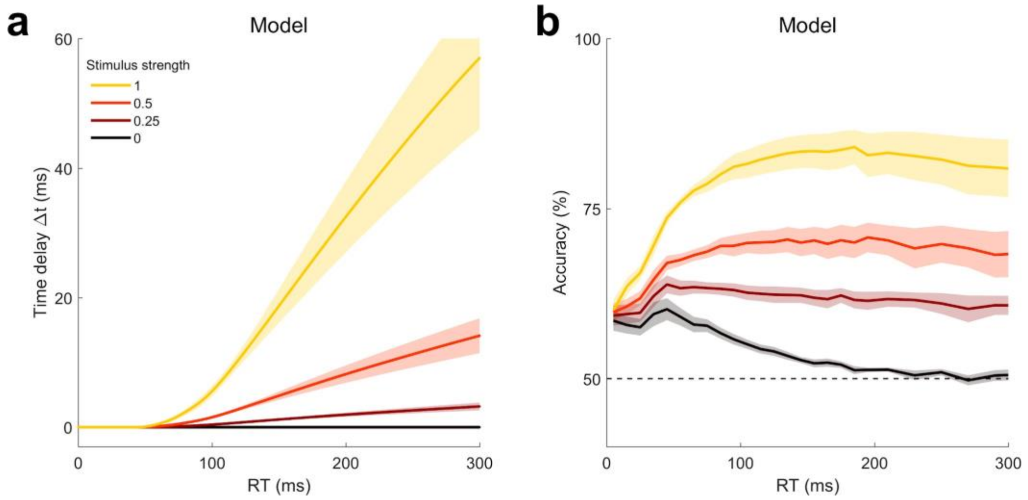


**Supplementary Figure 6. PSIAM simulations for expectation-biased trials. a.** Time delay curves. **b.** Tachometric curves. Legend as in Fig. 3d-e of the main manuscript, but for biased trials. The PSIAM simulations are qualitatively similar to the experimental data for biased trials (Supplementary Fig. 3b,d). All data are presented as mean values ± s.e.m. Source data are provided as a Source Data file.

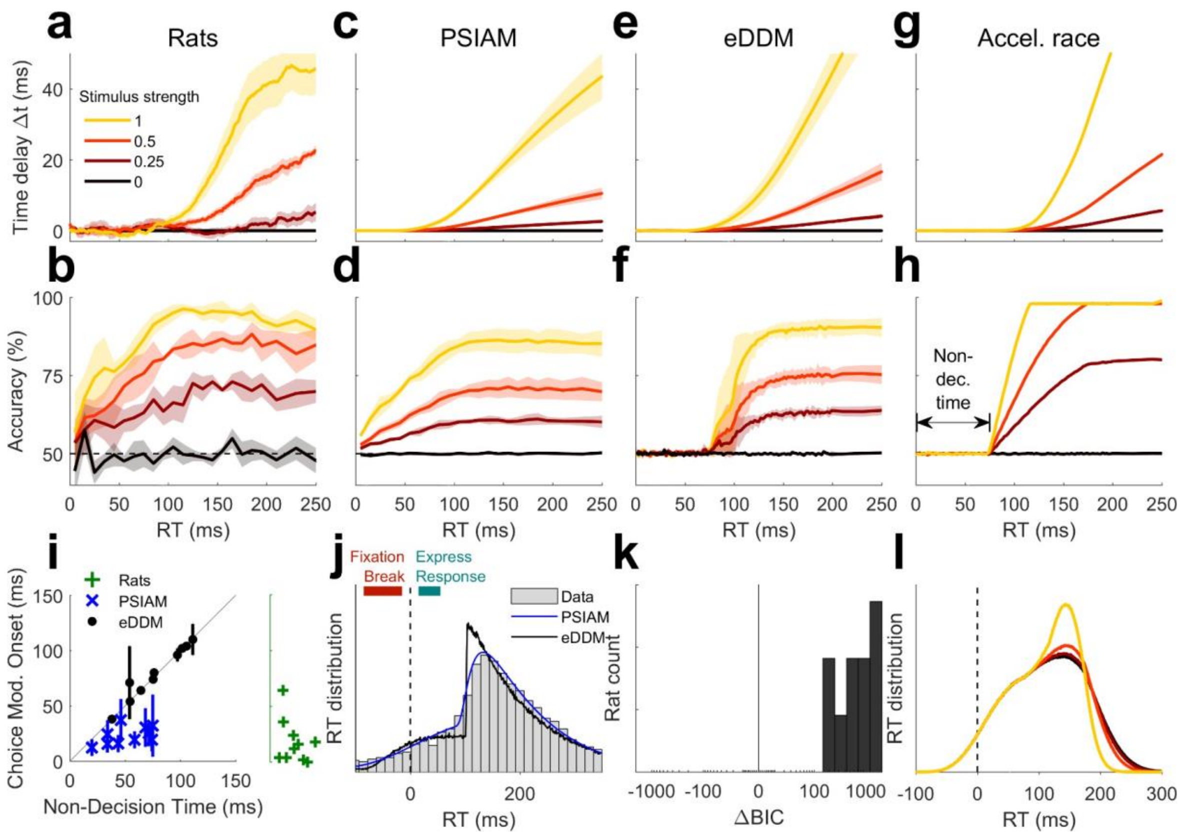


**Supplementary Figure 7. Model comparison. a-f,** Legend as in Figs. 2b,d and 3d-e,g-h of the main manuscript, respectively. Lines: median across rats (Group 1, *n* = 10). Shaded areas: median absolute deviation. **g-h,** Legend as in Fig. 3g-h of the main manuscript for an example simulation of the accelerated race model (Supplementary Methods). Lines: mean across realizations; s.e.m. smaller than line width. **i,** Temporal onset of the modulation of rat choices by stimulus, versus estimated non-decision time for PSIAM (blue tilted crosses) and eDDM (black solid circles). Each symbol represents a simulated animal. Choice modulation onset was defined as the shortest time at which the accuracy difference between strongest and weakest stimulus trials (stim. strengths *s*=1 and 0, respectively) became significant ($p<0.01$, two-sided paired t-test, 5-ms time bins). Choice modulation onset for experimental data is presented on the right (green crosses). **j,** RT distribution for an example rat (gray bars; rat #14) and model fits for PSIAM (blue line) and eDDM (black line) for stimulus strength *s*=0.25. Vertical dashed line denotes stimulus onset. **k,** Distribution of differences in Bayesian information criterion (ΔBIC) between eDDM and PSIAM for rat Group 1. Positive values indicate that the PSIAM provides a better account of experimental RT distribution. **l,** Simulated example RT distribution for the accelerated race model. Source data are provided as a Source Data file.

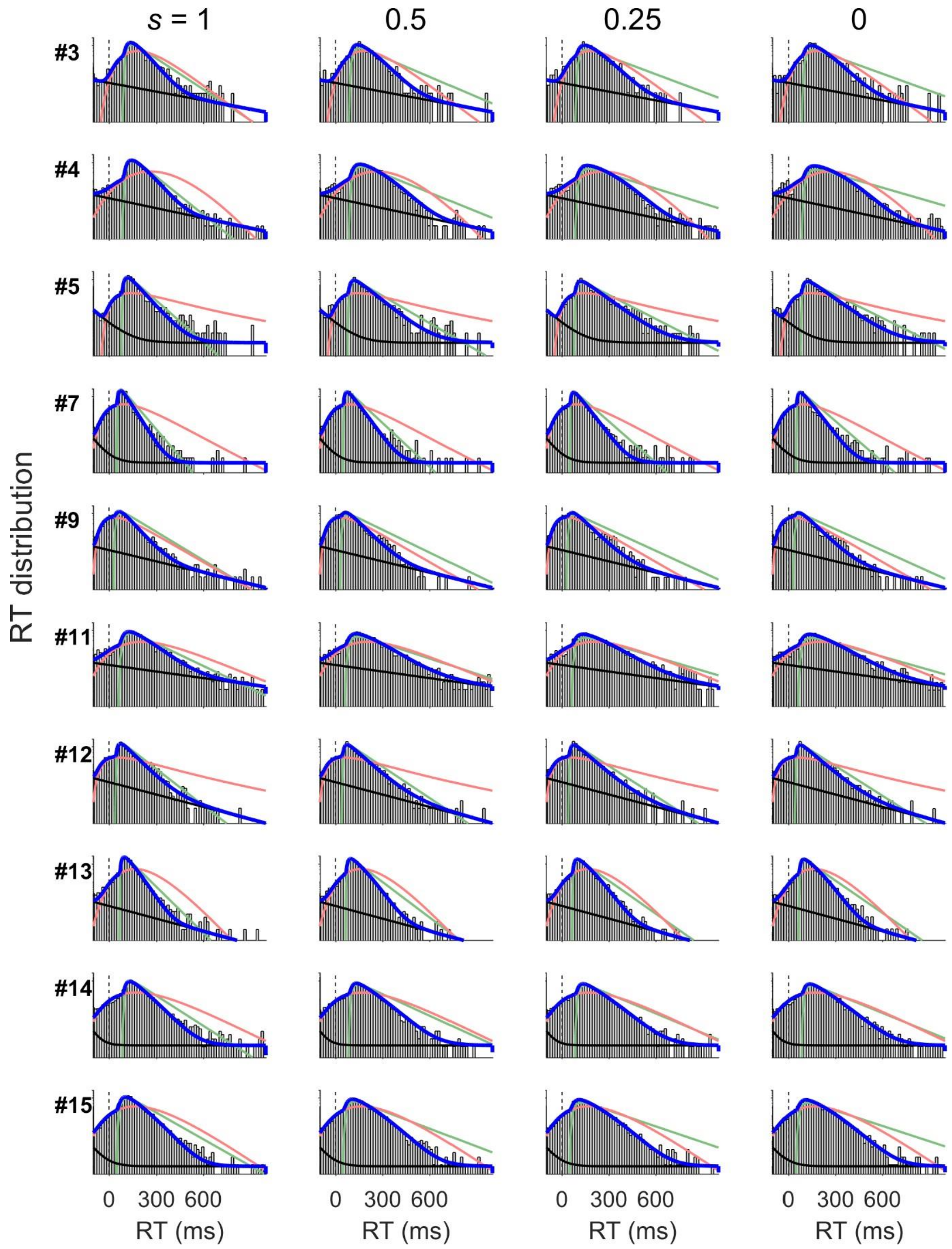


**Supplementary Figure 8. Distribution of long reaction times.** Legend as in Fig. 4a of the main manuscript. RT distributions in logarithmic scale (gray bars) and model fit (PSIAM, blue line; proactive responses, red; reactive responses, green; contaminants, black) for each rat in Group 1 (*n* = 10 rows, numeric label is rat index), and for each stimulus strength *s* (columns). Source data are provided as a Source Data file.

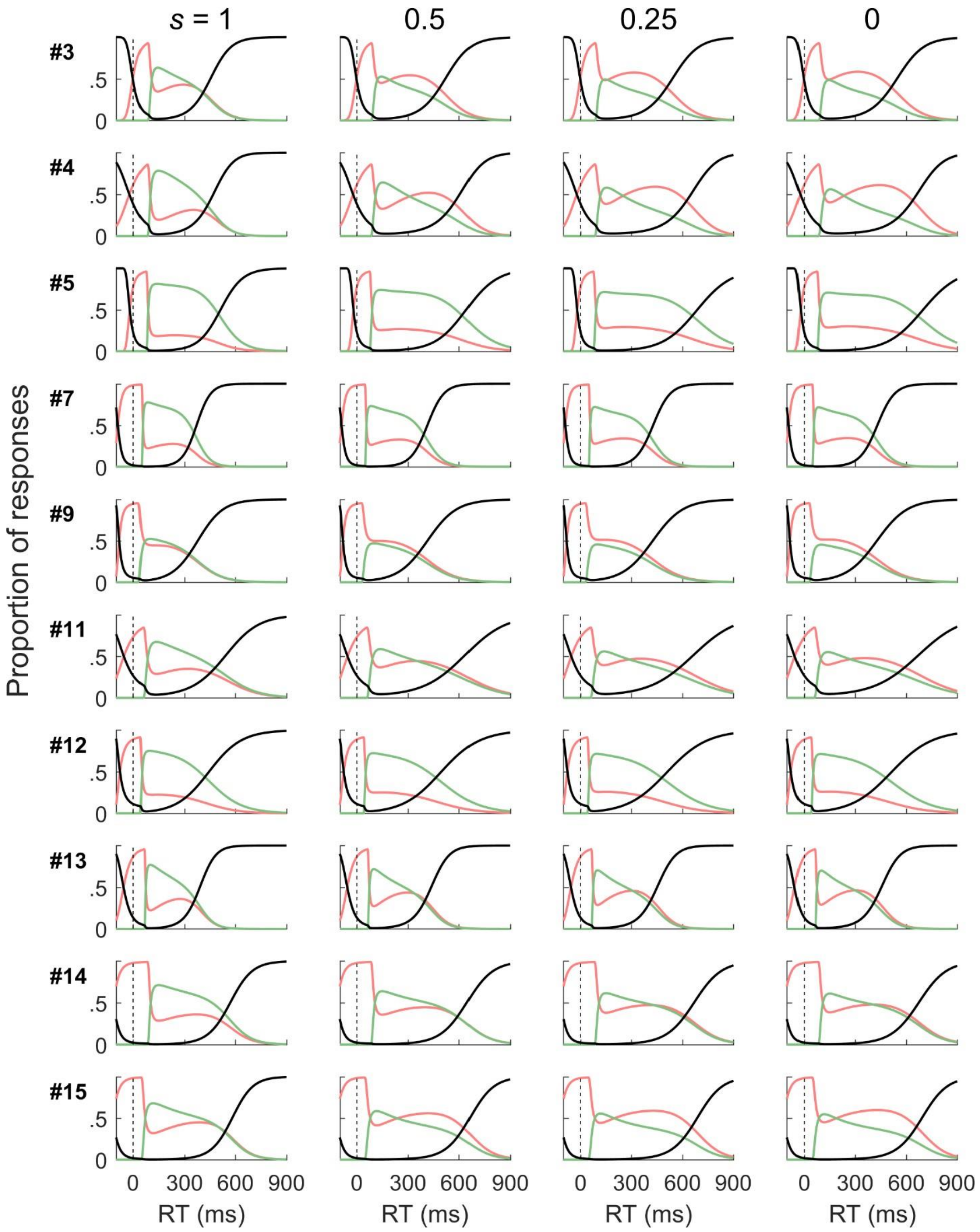


**Supplementary Figure 9. Proportion of responses.** Legend as in Fig. 4b of the main manuscript. Proportion of proactive (red), reactive (green) and contaminant (black) responses vs. RT for each rat in Group 1 ($n$ = 10 rows, numeric label is rat index), and for each stimulus strength $s$ (columns). Source data are provided as a Source Data file.

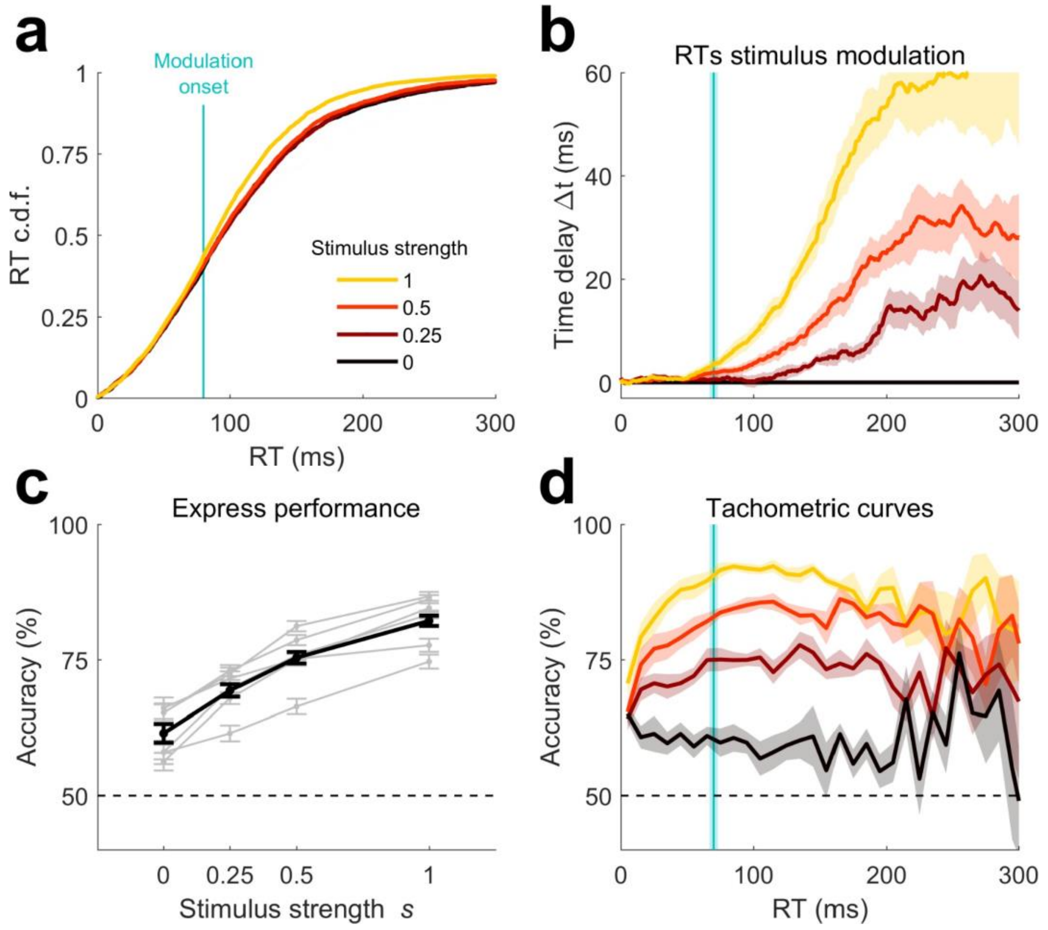


**Supplementary Figure 10. Decoupled reaction times and choices for express responses in the laterality task for unbiased and biased trials.** Legend as in Fig. 2 of the main manuscript, but for rats performing the laterality task instead of the frequency discrimination task, and for all trials. RT cumulative distribution functions (RT cdf, rat #46) (a), and time delay curves (b) for the laterality task are qualitatively similar to those for both unbiased and biased trials (Figs. 2a-b; Supplementary Fig. 3a-b, respectively). Accuracy in express responses (c) and tachometric curves (d) for the laterality task, unbiased and biased trials, are qualitatively similar to those of biased trials in frequency discrimination task (Supplementary Fig. 3c-d). All data points, data lines, error bars and error bands in panels b-d represent mean values ± s.e.m. across rats in Group 2 (*n* = 6). Source data are provided as a Source Data file.

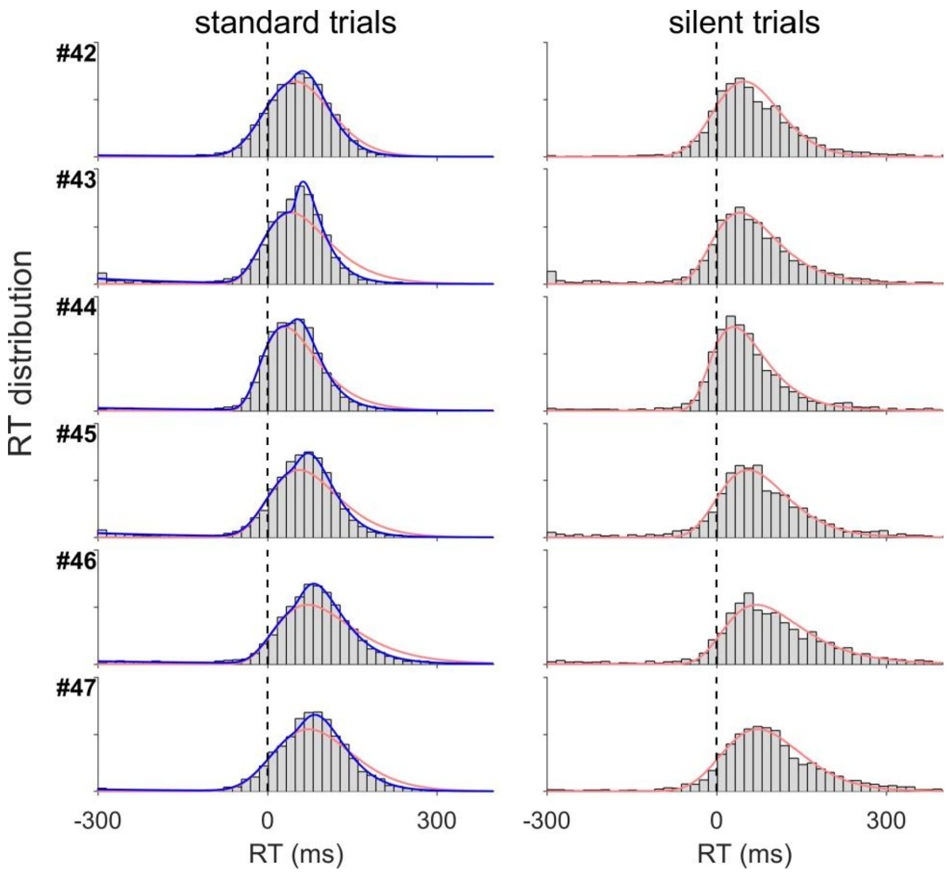


**Supplementary Figure 11. Reaction times in silent sessions.** Legend as in Fig. 5b,c of the main manuscript. Left column: RT distributions (gray bars) and model fit (PSIAM, blue; proactive responses, red) for standard trials for each rat in Group 2 (*n* = 6 rows, numeric label is rat index). Only standard trials with maximum stimulus strength (*s*=1) are shown. Right column: RT distributions (gray bars) and model predictions (proactive responses from the fit to standard RTs, red). Source data are provided as a Source Data file.

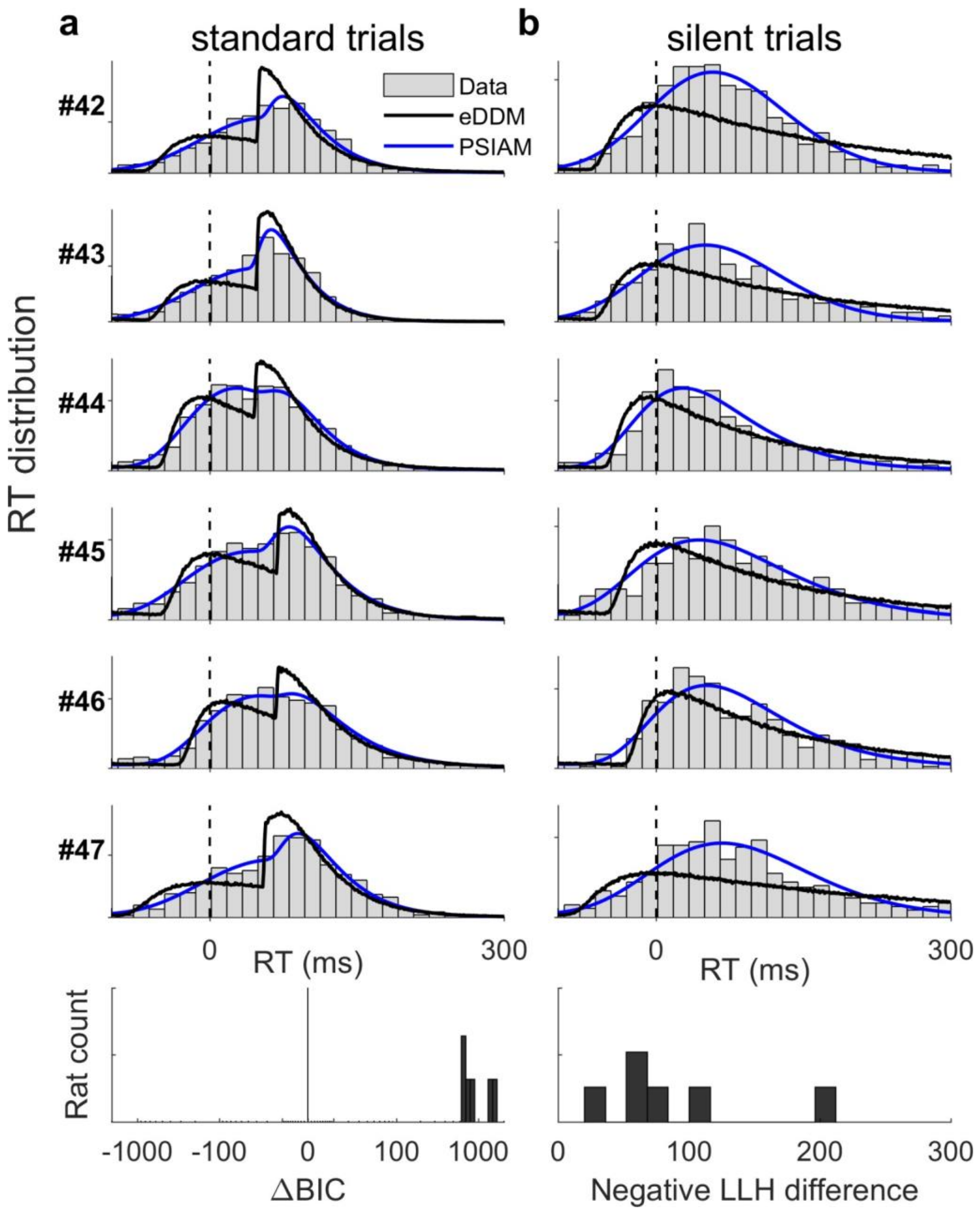


**Supplementary Figure 12. PSIAM and eDDM in silent sessions. a,** Legend as in Fig. 5b of the main manuscript. RT distributions (gray bars) and model fits for eDDM (black line) and PSIAM (blue line) fitted to unbiased, standard trials for each rat in Group 2 (*n* = 6 rows, numeric label is rat index). Last row: BIC difference between eDDM and PSIAM. **b,** Legend as in Fig. 5c of the main manuscript for eDDM (black line) and PSIAM (blue line) predictions for unbiased, silent trials. Last row: Negative log-likelihood model difference. Positive values indicate better predictions of silent RT distributions by the PSIAM. Source data are provided as a Source Data file.

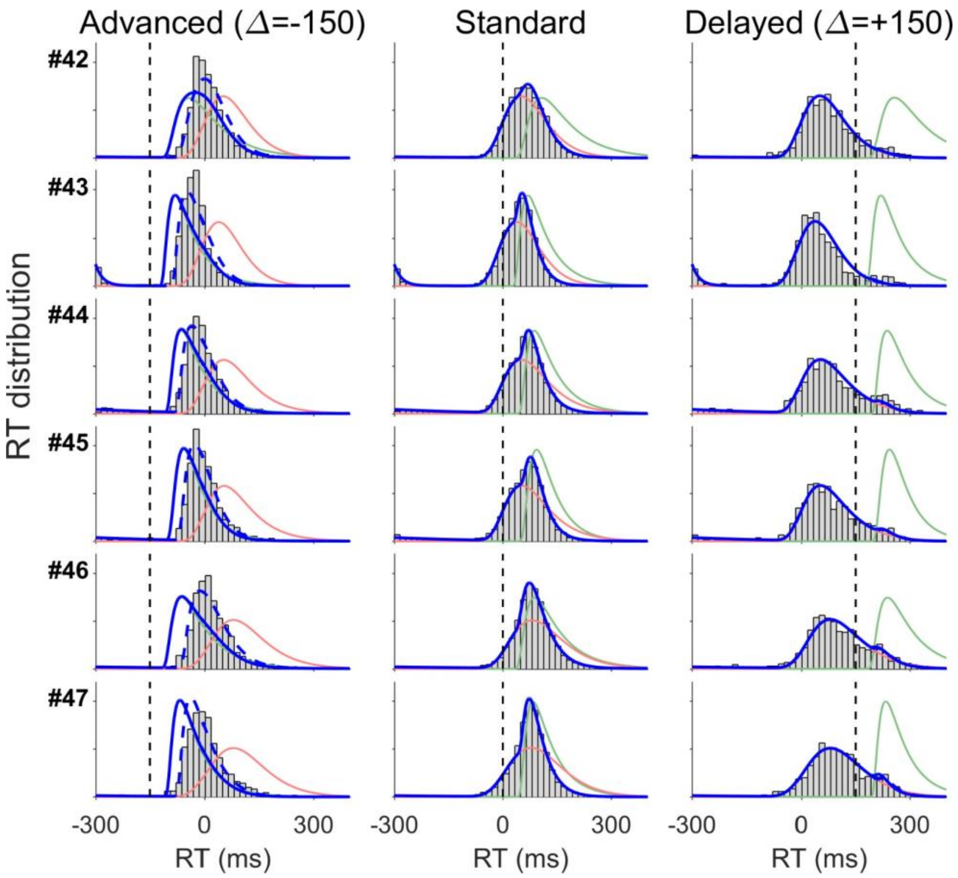


**Supplementary Figure 13. Reaction times in sessions with advanced and delayed stimulus onset (Group 2).** Legend as in Fig. 5d-e of the main manuscript. RT distributions (gray bars) and model fits and predictions for each rat in Group 2 (*n* = 6 rows, numeric label is rat index). Left column: RT distribution for advanced stimulus trials (gray bars), model prediction (solid blue line), corrected prediction (dashed blue), proactive responses from the fit to standard RTs (red), and reactive responses from the standard fit, shifted $\Delta$ = -150 ms (green). Center column: standard trials (gray bars), model fit (blue), proactive responses (red), and reactive responses (green). Right column: delayed stimulus trials (gray bars), model prediction (blue), proactive responses from the standard fit (red), and reactive responses from the standard fit, shifted $\Delta$ = +150 ms (green). For standard trials, only trials with maximum stimulus strength (*s*=1) are shown. The stimulus strength of all advanced and delayed stimulus trials was set to maximum (*s*=1, see Methods in main manuscript). Delay $\Delta$ in column heading is expressed in ms. Source data are provided as a Source Data file.

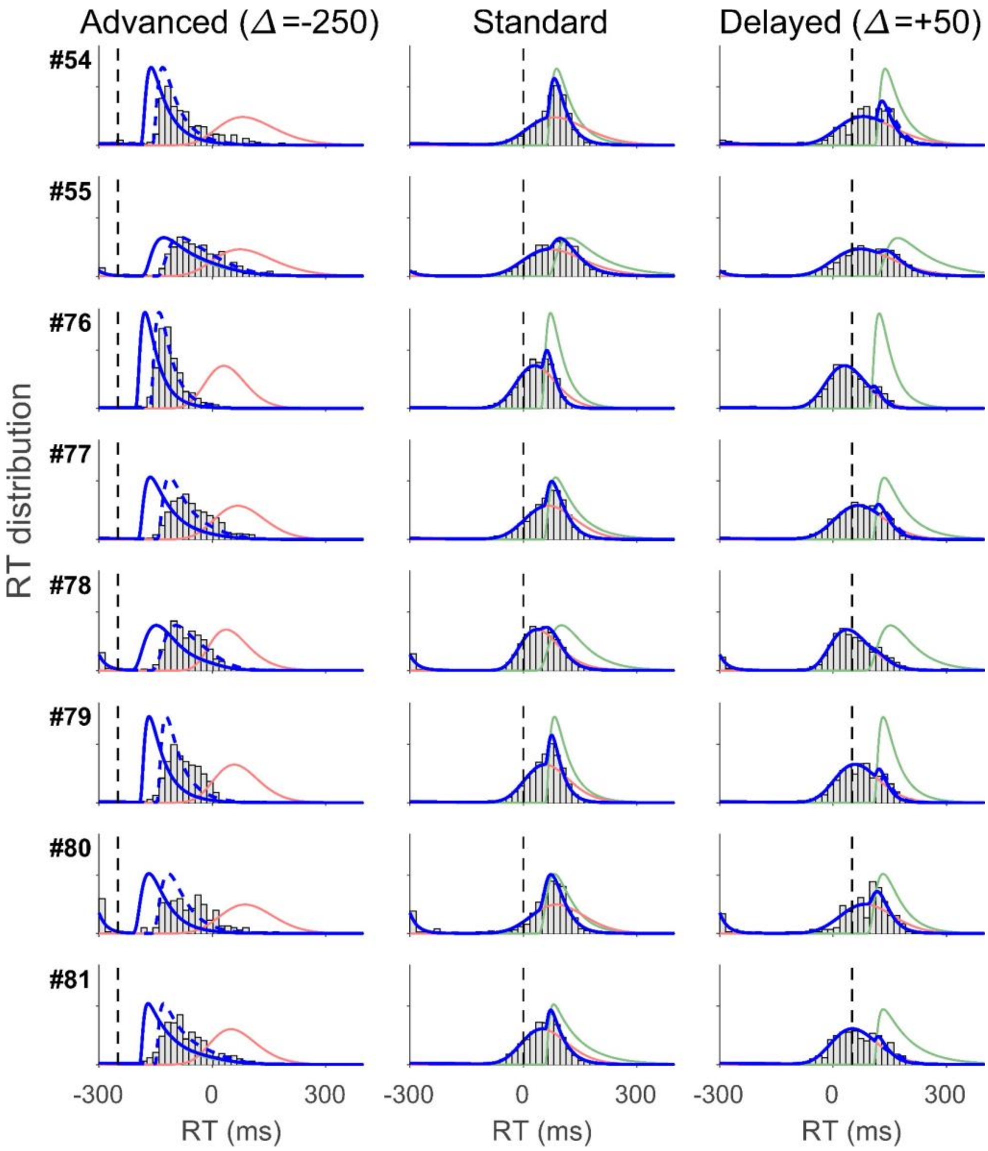


**Supplementary Figure 14. Reaction times in sessions with advanced and delayed stimulus onset (Group 3).** Legend as in Fig. 5d-e of the main manuscript and in Supplementary Fig. 13. RT distributions (gray bars) and model fits and predictions for each rat in Group 3 ($n$ = 8 rows, numeric label is rat index). Left column: RT distribution for advanced stimulus trials (gray bars), model prediction (solid blue line), corrected prediction (dashed blue), proactive responses from the fit to standard RTs (red), and reactive responses from the standard fit, shifted $\Delta$ = -250 ms (green). Center column: standard trials (gray bars), model fit (blue), proactive responses (red), and reactive responses (green). Right column: delayed stimulus trials (gray bars), model prediction (blue), proactive responses from the standard fit (red), and reactive responses from the standard fit, shifted $\Delta$ = +50 ms (green). For standard trials, only trials with maximum stimulus strength ($s$=1) are shown. The stimulus strength of all advanced and delayed stimulus trials was set to maximum ($s$=1, see Methods in main manuscript). Delay $\Delta$ in column heading is expressed in ms. Source data are provided as a Source Data file.

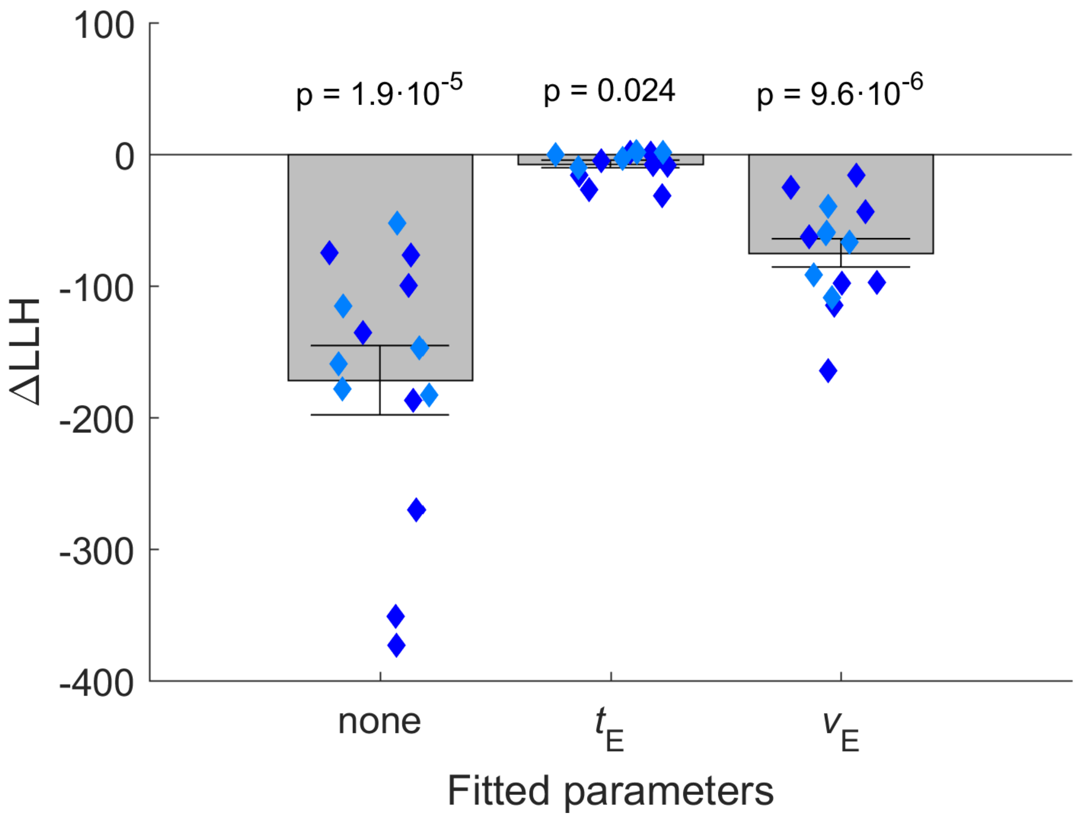


**Supplementary Figure 15. Marginal model evidence comparison for advanced and delayed stimulus trials.** Four models are compared for advanced and delayed stimulus trials: (1) PSIAM with parameters estimated from standard trials' RTs; (2) PSIAM where EA latency ($t_E$) is fitted for each delay condition (all other parameters taken from standard trials estimates); (3) PSIAM where EA drift ($v_E$) is fitted for each delay condition (all other parameters taken from standard trials estimates); and (4) PSIAM where both EA latency and drift are fitted to each delay condition; i.e. estimated parameters: none, $t_E$, $v_E$, and $\{t_E, v_E\}$, respectively. The marginal evidence was computed as the mean likelihood of each model after marginalizing over the fitted parameters (computing LLH for parameters in a grid with small step size), and then compared in logarithmic units to the marginal evidence of the $\{t_E, v_E\}$ model. Negative values indicate that the full model (i.e. with both EA latency and drift fitted) is favored by the data. Diamonds: individual rats (Group 2: light blue, $n$ = 6; Group 3: dark blue, $n$ = 8); bars: mean across rats; error bars: s.e.m.; p-values: two-tailed pairwise t-test comparisons with full model. Source data are provided as a Source Data file.

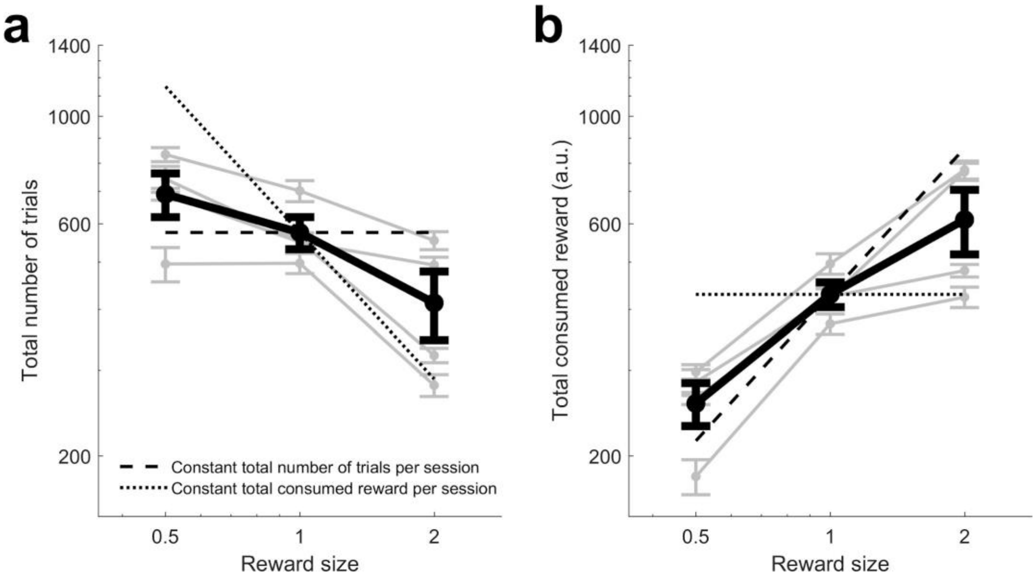


**Supplementary Figure 16. Reward size impact on session length and reward consumption. a-b,** Total number of trials per session (a) and total consumed reward per session (b) vs. reward size (Group 4, *n* = 4). Thick lines: group average; thin lines: individual rats; error bars: s.e.m.; dashed lines: constant total number of trials across reward sizes; dotted lines: constant consumed reward across reward sizes. Notice that, for smaller reward sizes, rats mildly increased the total number of trials, which barely compensated for the lower reward consumption. For larger rewards however, half of the rats performed the standard number of trials, which doubled their total consumed reward, whereas the other half reduced the total number of trials while maintaining a standard total reward consumption. Source data are provided as a Source Data file.

| Rat Group 1 (mean ± s.e.m.) | Parameters | | Unbiased trials | Biased trials |
|---|---|---|---|---|
| **Action Initiation process** | Drift | Intercept $□_{A0}$ | 5.2 ± 0.5 $s^{-1}$ | 7.4 ± 0.5 $s^{-1}$ |
| | | Trial-index weight $□_{trial}$ | (-2.5 ± 0.5)·$10^{-3}$ $s^{-1}$ | (-6 ± 1.3)·$10^{-3}$ $s^{-1}$ |
| | Go bound $□_A$ | | 3.0 ± 0.7 | 2.2 ± 0.3 |
| | Latency $t_A$ | | -50 ± 70 ms | 90 ± 30 ms |
| **Evidence Accumulation process** | Drift | stimulus-strength weight $□_E$ | 2.8 ± 0.2 $s^{-1}$ | 2.7 ± 0.3 $s^{-1}$ |
| | Decision bounds ± $□_E$ | | 0.45 ± 0.03 | 0.52 ± 0.03 |
| | Latency $t_E$ | | 52 ± 6 ms | 47 ± 5 ms |
| | Starting offset $z_E$ | | 0 (fixed) | (0.21 ± 0.04)· $□_E$ |
| **Contaminants** | Proportion $c$ | | 0.18 ± 0.02 | 0.038 ± 0.006 |
| | Exp-uniform mixture $d$ | | 0.97 ± 0.014 | 1 |
| | Exp. time constant □ | | 9 ± 3 $s^{-1}$ | 1.5 ± 0.14 $s^{-1}$ |

**Supplementary Table 17. PSIAM parameter estimates.** Parameter values (mean ± s.e.m. across animals) extracted from the fit of PSIAM to RTs for each animal in Group 1 ($n$ = 10).

| Rat Group 1 (mean ± s.e.m.) | Parameters | Unbiased trials |
|---|---|---|
| **DDM** | Pre-stimulus internal noise [illegible]$_I$ | $0.51 \pm 0.02$ s$^{-1}$ |
| | Pre-stim. response latency $t_{latency}$ | 210 ± 14 ms |
| | Decision bounds ± [illegible] | 0.36 ± 0.02 |
| | Drift stimulus weight [illegible]$_E$ | $3.2 \pm 0.4$ s$^{-1}$ |
| | Non-decision time $t_E$ | 78 ± 8 ms |
| **Contaminants** | Proportion *c* | 0.18 ± 0.02 |
| | Exp-uniform mixture *d* | 0.98 ± 0.01 |
| | Exp. time constant [illegible] | $8 \pm 2$ s$^{-1}$ |

**Supplementary Table 18. eDDM parameter estimates.** Parameter values (mean ± s.e.m. across animals) extracted from the fit of eDDM to unbiased trial RTs for each animal in Group 1 ($n = 10$).

## Supplementary Methods

### Acoustic stimulus

In the two acoustic tasks used, the stimulus $S_k(t)$ in the $k$-th trial was created by simultaneously playing two amplitude modulated (AM) sounds $T_R(t)$ and $T_L(t)$ :

[illegible] = [1 + [illegible]( [illegible] + φ)][[illegible] + [illegible]] (1)

The AM frequency was $f_{AM}$=20 Hz and the phase delay $\varphi = 3\pi/2$ made the envelope zero at $t = 0$. In the frequency discrimination task, $T_L(t)$ and $T_R(t)$ were pure tones with frequencies 6.5 kHz and 31 kHz, respectively, played simultaneously in the two speakers. In the level discrimination task they were broadband noise played either from the Left or the Right speaker, respectively. The amplitudes of the sounds $s_L(t)$ and $s_R(t)$ were separately calibrated at 65 dB (Group 2) or 70 dB (Group 1) using a free-field microphone (Med Associates Inc,

ANL-940-1). Sounds were delivered through generic electromagnetic dynamic speakers (Group 1: STAX, SRS-2170; Group 2: ZT-026 YuXi) located on each side of the chamber.

### Stimulus Sequence

A Markov chain generated the sequence of stimulus category $c_k$= {-1,1}, that determined whether the reward in the *k*-th trial was available in the Left or the Right port respectively. The stimulus category $c_k$ set which of the two sounds $T_L(t)$ and $T_R(t)$ composing each stimulus was dominant, which ultimately determined the statistics of the sound amplitudes [illegible] and [illegible] (Supp. Eq. 1) as described below. In each trial, independently of $c_k$, the stimulus strength $s_k$ was also randomly sampled from 4 possible values: 0, 0.25, 0.5 and 1. Stimulus strength $s_k$ defined the relative weights of the dominant and non-dominant sounds: for example, when $s_k$ = 1 only the dominant sound was played (i.e. easiest trials) whereas when $s_k$ = 0 the two sounds had on average the same amplitude (i.e. hardest trials). The stimulus evidence was defined in each trial as the combination $e_k = c_k * s_k$ . The value of $e_k$ determined the p.d.f. from which the instantaneous evidence $S_{k,f}$ was drawn in each frame *f* (i.e. in each 50 ms AM-envelope cycle): when $e_k$:=±1 the p.d.f. was [illegible] = δ([illegible] ∓ 1) (i.e. a Dirac delta p.d.f.) whereas when $e_k \in$ (-1,1), it was a stretched Beta distribution with support [-1,1], mean equal to $e_k$ and variance equal to 0.06. Finally, the amplitudes [illegible] and [illegible] of the two AM envelopes were obtained using [illegible] = (1 + [illegible])/2 and [illegible] = (1 − [illegible])/2 with *f* referring to the frame index that corresponds to the time *t*. With this choice the sum of the two envelopes was constant in all frames $a^L_k(t)+a^R_k(t)=1$.

We introduced two types of serial correlations in the stimulus trial sequences using blocks of 200 consecutive trials, interleaved within a session. In the repeating trial block, the probability to repeat the previous stimulus category was larger than chance ($p_{rep}$= 0.7); in the alternating trial block, the probabilities were reversed ($p_{alt}$= 0.8). We have shown previously that rats performing this task develop a history-dependent bias to repeat or alternate their response, and that this bias vanishes after error trials [1] (Supplementary Fig. 1). Rats in Groups 2 and 3 performed additional sessions where the stimulus probability was manipulated: in the left-enhanced block, the probability of left stimulus category was larger than chance ($p_{left}$= 0.8); in the right-enhanced block, the probabilities were reversed ($p_{right}$= 0.8).

### Model fit for biased trials

For biased trials, the non-zero EA initial offset captured the animal's trial-dependent expectation of the next rewarded side as $Z_E = z_E \cdot b_k$ , where the magnitude of the expectation $z_E$ was fitted to the data, whereas the trial-dependent category of the expected rewarded side $b_k$ = ±1 was set by the stimulus sequence and the type of serial correlations. For repeating and alternating blocks, the expected rewarded side depended on the category of the previous response $R_{-1}^+$ and on the block type ($B$ = +1 or -1, respectively), so that $b_k$ =

$B{\cdot}R_{-1}^{+}$ . For left and right-enhanced blocks in Groups 2 and 3, the expected rewarded side was equal to the block type $b_k = B$.

**Model prediction for choice data in biased trials**

For PSIAM simulations of biased trials, we included a small trial-to-trial variability in the EA starting offset (otherwise choices for very short RTs in biased trials would always be determined by the side of $Z_E$, i.e. the expectation, unlike what is observed in rats). The distribution of starting point was thus taken as a Beta distribution (stretched to cover the full domain of the EA process [- [illegible] E, [illegible] E]). The parameters of the distribution were set such that it had mean $z_E$ and standard deviation equal to 10% of the interbound distance between bounds (i.e. 0.2· [illegible] E).

**Model comparison**

We compared how the PSIAM and extended DDM accounted for the distributions of rat RTs in unbiased standard trials using the Bayesian Information Criterion (BIC):

[illegible] = [illegible]·[illegible] ([illegible]) − 2·[illegible], (2)

where $k$ is the number of parameters, $N$ the number of trials, and LLH the log-likelihood of the model at estimated parameters. The difference in BIC between eDDM and PSIAM is then interpreted as a metric of relative model performance, where positive values indicate more evidence towards PSIAM (Supp. Figs. 7k and 12a). We also compared the ability of each model, fitted on standard trials, to predict RT distributions in silent catch trials. We computed the Log-Likelihood (LLH) of silent trials RTs for the previously fitted models, where a negative LLH difference provides evidence towards PSIAM (Supp. Fig. 12b).

**Accelerated-race model**

The accelerated race model [2] assumes a ballistic race between Left and Right motor response plans initiated prior to stimulus onset. The race is modulated by stimulus identity after stimulus onset, which accelerates the target motor plan and decelerates the distractor. Choice and RT are set by the first of the two motor plans reaching a fixed threshold. We simulated the model (~$10^8$ trial realizations) exactly as in Stanford et al. with one exception: for proper comparison with PSIAM, the signed stimulus strength $S$ determined the acceleration rate:

[illegible] = [illegible] for [illegible] < [illegible] and [illegible] < θ

τ · [illegible] = ([illegible] + [illegible]·[illegible]) − [illegible] for [illegible] < [illegible] < τ and [illegible] < θ (3)

where *x(t)* is the motor plan for either Left or Right response, with initial constant rate $r_{X,0}$ (sampled from a bivariate normal distribution of mean *r*, variance □$_r^2$ and correlation □$_S$); *t* is the stimulus onset time; □ the threshold; $r_m$ the intercept of the final rate; $r_s$ the stimulus weight of the final rate; *S* the signed stimulus strength (which is positive for target and negative for distractor, with a probability $p_{swap}$ of swapping signs); and □ the acceleration time window. The race onset time is sampled from a normal distribution with zero mean and standard deviation of 10ms; once the threshold is hit, the response is initiated with latency $t_{ND}$. We set the parameter values to: $r$ = 2200 $s^{-1}$, □$_r$ = 1100 $s^{-1}$, □ = -0.5, $t_S$ = 0.3 s, □ = 1000, $r_m$ = 4400 $s^{-1}$, $r_S$ = 8800 $s^{-1}$, □ = 0.1 s, and $t_{ND}$ = 0.075 s; and simulated a total of $10^8$ trials (Supp. Fig. 7g-h,l).


1. Hermoso-Mendizabal, A. *et al.* Response outcomes gate the impact of expectations on perceptual decisions. *Nat. Commun.* **11**, 1057 (2020).
2. Stanford, T. R., Shankar, S., Massoglia, D. P., Gabriela Costello, M. & Salinas, E. Perceptual decision making in less than 30 milliseconds. *Nature Neuroscience* vol. 13 379–385 (2010).
