# Supplementary material for: Proactive and reactive accumulation-to-bound processes compete during perceptual decisions
Source: Nat Commun. 2021 Dec 8;12:7148. doi: 10.1038/s41467-021-27302-8 (PMC8655090; doi:10.1038/s41467-021-27302-8)
Supplement: Supplementary file 3 — Reporting Summary [file 41467_2021_27302_MOESM3_ESM.pdf]

nature research

Corresponding author(s): Alexandre Hyafil
Last updated by author(s): Sep 29, 2021

Reporting Summary

Nature Research wishes to improve the reproducibility of the work that we publish. This form provides structure for consistency and transparency in reporting. For further information on Nature Research policies, see our Editorial Policies and the Editorial Policy Checklist.

Statistics

For all statistical analyses, confirm that the following items are present in the figure legend, table legend, main text, or Methods section.

n/a	Confirmed	
☐	☒	The exact sample size (*n*) for each experimental group/condition, given as a discrete number and unit of measurement
☐	☒	A statement on whether measurements were taken from distinct samples or whether the same sample was measured repeatedly
☐	☒	The statistical test(s) used AND whether they are one- or two-sided<br>*Only common tests should be described solely by name; describe more complex techniques in the Methods section.*
☒	☐	A description of all covariates tested
☐	☒	A description of any assumptions or corrections, such as tests of normality and adjustment for multiple comparisons
☐	☒	A full description of the statistical parameters including central tendency (e.g. means) or other basic estimates (e.g. regression coefficient) AND variation (e.g. standard deviation) or associated estimates of uncertainty (e.g. confidence intervals)
☐	☒	For null hypothesis testing, the test statistic (e.g. *F*, *t*, *r*) with confidence intervals, effect sizes, degrees of freedom and *P* value noted<br>*Give P values as exact values whenever suitable.*
☐	☒	For Bayesian analysis, information on the choice of priors and Markov chain Monte Carlo settings
☒	☐	For hierarchical and complex designs, identification of the appropriate level for tests and full reporting of outcomes
☒	☐	Estimates of effect sizes (e.g. Cohen's *d*, Pearson's *r*), indicating how they were calculated

Our web collection on statistics for biologists contains articles on many of the points above.

Software and code

Policy information about availability of computer code

Data collection	The experimental data were collected using the behavioral setup (Island Motion, NY). For rat Group 1, this setup was controlled by a custom software developed in Matlab (version R2014a, Mathworks, Natick, MA), based on the open-source BControl framework (http://brodylab.princeton.edu/bcontrol). For Groups 2-4, the behavioral setup was controlled by BPod electronic boards (by Sanworks) and the task was run using the Python-based open software package PyBPod (http://pybpod.com/).
Data analysis	The data analysis was performed using custom Matlab code (version R2019a, Mathworks, Natick, MA), and a customized version of PyDDM (https://pyddm.readthedocs.io/en/stable/; Spyder, Python 3.7). Custom code is available in the public repository: https://osf.io/3qe59/.

For manuscripts utilizing custom algorithms or software that are central to the research but not yet described in published literature, software must be made available to editors and reviewers. We strongly encourage code deposition in a community repository (e.g. GitHub). See the Nature Research guidelines for submitting code & software for further information.

Data

Policy information about availability of data

All manuscripts must include a data availability statement. This statement should provide the following information, where applicable:

- Accession codes, unique identifiers, or web links for publicly available datasets
- A list of figures that have associated raw data
- A description of any restrictions on data availability

Data generated during the study is available in a public repository (https://osf.io/3qe59/).

Field-specific reporting

Please select the one below that is the best fit for your research. If you are not sure, read the appropriate sections before making your selection.

☒ Life sciences ☐ Behavioural & social sciences ☐ Ecological, evolutionary & environmental sciences

For a reference copy of the document with all sections, see nature.com/documents/nr-reporting-summary-flat.pdf

Life sciences study design

All studies must disclose on these points even when the disclosure is negative.

Sample size	The sample size for the first group of 10 rats was grounded on Hermoso-Mendizabal et al., Nat. Comm. (2020). For the second, third and fourth groups, of 6, 8 and 4 rats respectively, group sizes were set based on (1) the variability across rats observed in previous experiments, (2) the total number of trials required to resolve the distribution of reaction times with sufficient accuracy for each tested condition and (3) the experimental time required to acquire those data.
Data exclusions	No data were excluded from the analyses.
Replication	We obtained the main results with a first group of 10 rats . These results where replicated and model predictions were tested with a second group of 6 rats. A third group of 8 rats replicated all previous results. Results obtained by the fourth group of 4 rats were consistent with all previous results.
Randomization	The animals of each group were randomly selected. No criteria based on task performance or other task-relevant variables were used to assign groups other than the condition that animals had learned the task.
Blinding	Blinding was not applicable because there were no comparisons across groups (all analyses are performed within each group)

Reporting for specific materials, systems and methods

We require information from authors about some types of materials, experimental systems and methods used in many studies. Here, indicate whether each material, system or method listed is relevant to your study. If you are not sure if a list item applies to your research, read the appropriate section before selecting a response.

Materials & experimental systems

n/a	Involved in the study	
☒	☐	Antibodies
☒	☐	Eukaryotic cell lines
☒	☐	Palaeontology and archaeology
☐	☒	Animals and other organisms
☒	☐	Human research participants
☒	☐	Clinical data
☒	☐	Dual use research of concern

Methods

n/a	Involved in the study	
☒	☐	ChIP-seq
☒	☐	Flow cytometry
☒	☐	MRI-based neuroimaging

Animals and other organisms

Policy information about studies involving animals; ARRIVE guidelines recommended for reporting animal research

Laboratory animals	Animals were male Long-Evans rats (n = 26, 350-650g; Charles River). They had 8-10 weeks when the experiment started, which lasted between 2 and 8 months.
Wild animals	Not used.
Field-collected samples	No field-collected samples were used in the study.
Ethics oversight	The ethics committee was the Comité d'Experimentació Animal from University of Barcelona, Spain. The reference of the approved protocol was 390/14.

Note that full information on the approval of the study protocol must also be provided in the manuscript.
